# 

**Published:** 1916-10-28

**Authors:** 


### CONTENTS.

PAGE

**An Inspiring Address** ... ... ... ... ... ... 57

**The Notification of Venereal Diseases** ... ... 58

**Hospital and Institutional News** ... ... ... 59

Our Special Number—The Government and the Welsh Medical School—Gardelegen Horrors—The Success of a War-Time Appeal—The Reward of Administration in Liverpool—War Hospital Muddle at Ashford—Rawtenstall Military Hospital—A Remarkable Village Collection—The Chadwick Trust Public Lectures—Naval Philanthropy—The Removal of Scarlet Fever Patients—A Housing Proposal at Newcastle—Government Deductions during Hospital Treatment—Exploitation of V.A.D.s by Nursing Homes—The Segregation of Wounded Canadians—A Hospital Nurse's Claim for a Legacy—An Unusual Test—A Problem of National Health in Greater London—No Economy in Health Services—Polling Booths in Voluntary Hospitals—This Week's Drug Market.

**Hospital Policy in America: Problems of the Future** ... ... ... ... ... ... ... 63

By Winford H. Smith, M.D., Superintendent, The Johns Hopkins Hospital, Baltimore, Md.

**Passing Events and Topics** ... ... ... ... ... 68

**Mr. F. N. Clare Melhado** ... ... ... ... ... 69

A Valued Hospital Administrator.

PAGE

**Parliament and the Profession** ... ... ... ... 70

**Names and Things** ... ... ... ... ... ... 71

The Universal Craving for Euphemisms.

**Lessening the Tedium of Hospital Life** ... ... 72

The Birmingham System at Work.

**The Matrons' and Sisters' Department** ... 73

District Nurses and their Work.

Round the Hospitals.

The College of Nursing—New Conditions of Registration—Patriotism in Nurse-Training Schools—Presentation to Miss Girdlestone—Local Ladies' Committees, T.F.N.S.—Almoners' Work at St. Thomas's.

**The College of Nursing, Limited** ... .. ... 75

Conditions of Registration Applicable during the Period of Grace to Existing Nurses.

**Gifts to Hospitals** ... ... ... ... ... ... 75

**Matrons' and Sisters' Appointments** ... ... 76

**The Book Market** ... ... ... ... ... ... 76

**"An Unfortunate Controversy at Leeds"** ... 76

**The Institutional Worker** ... .. (Suppl.) 1

The Supply of Comforts for Wounded and Warriors.

Question for October.

Questions Invited.

Enquiries and Answers.

